# BatTool: projecting bat populations facing multiple stressors using a demographic model

**DOI:** 10.1186/s12862-023-02159-1

**Published:** 2023-10-16

**Authors:** Ashton M. Wiens, Amber Schorg, Jennifer Szymanski, Wayne E. Thogmartin

**Affiliations:** 1 U.S. Geological Survey, Upper Midwest Environmental Sciences Center, La Crosse, WI 54603 USA; 2 U.S. Fish and Wildlife Service, Ecological Services, Illinois-Iowa Field Office, Moline, IL 61265 USA; 3 U.S. Fish and Wildlife Service, Division of Endangered Species, La Crosse Fish and Wildlife Conservation Office, Onalaska, WI 54650 USA

**Keywords:** Population ecology, Cave-hibernating bats, Wind energy, Demography, Matrix projection, Population viability analysis, Extinction risk, White-nose syndrome

## Abstract

Bats provide ecologically and agriculturally important ecosystem services but are currently experiencing population declines caused by multiple environmental stressors, including mortality from white-nose syndrome and wind energy development. Analyses of the current and future health and viability of these species may support conservation management decision making. Demographic modeling provides a quantitative tool for decision makers and conservation managers to make more informed decisions, but widespread adoption of these tools can be limited because of the complexity of the mathematical, statistical, and computational components involved in implementing these models. In this work, we provide an exposition of the BatTool R package, detailing the primary components of the matrix projection model, a publicly accessible graphical user interface (https://rconnect.usgs.gov/battool) facilitating user-defined scenario analyses, and its intended uses and limitations (Wiens et al., US Geol Surv Data Release 2022; Wiens et al., US Geol Surv Softw Release 2022). We present a case study involving wind energy permitting, weighing the effects of potential mortality caused by a hypothetical wind energy facility on the projected abundance of four imperiled bat species in the Midwestern United States.

## Introduction

Bats play an important ecological and agricultural role in controlling insect populations, but many bat species are currently experiencing population declines as they face multiple environmental stressors [1]. The U.S. Fish and Wildlife Service (USFWS) has already listed several cave-hibernating bat species as threatened or endangered under the Endangered Species Act (ESA) [2, 3]. White-nose syndrome (WNS), a deadly disease caused by the fungal pathogen *Pseudogymnoascus destructans*, has contributed most to the decimation of many populations of cave-hibernating bats [1] and is quickly spreading across North America [4–10]. In addition, wind energy developments threaten migratory bats, whose carcasses have been found at many wind turbine operations throughout the United States [11]. Estimated fatalities combined with demographic modeling suggest that bats will likely be impacted by increased wind energy development [12, 13]. These as well as other factors including habitat loss and fragmentation, human disturbance, flying hazards, insecticide use, and climate change motivate analyses of the current and future health of these species.

Conservation managers and decision makers frequently rely on population viability analysis (PVA) when assessing the future health of a population or species. Matrix projection models are a useful tool for conducting PVA, combining species-specific demography into a statistical framework capable of incorporating sources of uncertainty and producing probabilistic forecasts of population abundance and risk of extinction [14, 15]. These models can accommodate a diverse set of life histories and can be used to determine which life stages and vital rates contribute most to population health and future viability. In addition, these models are also used to evaluate the impacts of various stressors and management approaches, allowing PVA to inform effective decision strategies and have significant policy implications in environmental conservation. For example, [16] combined matrix projection models with estimates of wind turbine-caused bird fatalities to assess the relative vulnerability of raptor species to current and future wind energy. More generally, [17] developed a spatially explicit framework for evaluating population-level impacts of anthropogenic stressors on terrestrial wildlife. Widespread adoption of these mathematical and statistical tools in ecological decision making can, however, be limited by implementation and accessibility barriers. Making these models easily accessible to a wider audience can be achieved by creating an application with a graphical user interface (GUI), so the user can avoid writing code.

Matrix projection models have been developed to forecast population dynamics in bat populations facing environmental stressors [4, 15]. The BatTool [18] is a package in R [19] providing access to code and a GUI implementing the model developed in [15]. This statistical model and R package have allowed experts to use population projections under different scenarios as a tool when assessing the status of bats and the effects of possible future environmental stressors. USFWS and collaborators have used the BatTool in one analysis contributing to a species status assessment for three cave-hibernating bat species affected by WNS, *Myotis lucifugus*, *M. septentrionalis*, and *Perimyotis subflavus*, to help decide their conservation status under the ESA [20]. USFWS has also utilized the tool to assess mortality consequences when issuing Incidental Take Permits to entities pursuing wind energy development; see [21–23]. Currently, the existing BatTool R package and some of its dependencies are no longer maintained.

The purpose of our work is two-fold: we introduce the updated BatTool R package with web-enabled GUI [24, 25] and demonstrate use of the tool in a case study assessing the potential impacts of wind energy generation on bat populations and permitting decisions. The updated package has increased modeling functionality available to the user, and the new GUI is developed using the Shiny package [26] in R, which enables R users to easily create and distribute interactive web applications, including complex mathematical and statistical models [27]. To make the software more broadly available, the GUI is hosted publicly at https://rconnect.usgs.gov/battool [24, 25], obviating the need for users to maintain R locally while still reaping the benefits of the R programming language. Our case study provides an example of how the model and tool can be used for PVA, including quantifying the populations under investigation and the stressors affecting them. The documented process and results could be used as a template for future wind permitting analyses or more generally for assessing impacts of wind energy development or other stressors on bat populations.

In the Materials and methods section, we review the demographic model in the context of the updated BatTool R package and associated application. In the Results section, we provide details on the case study and present the outcomes. The Discussion section contains model assumptions and limitations.

## Materials and methods

One quantitative approach to population dynamics includes simulating the future population trajectory of a single population based on its current status and trend (population abundance and growth rate, respectively). Matrix projection models are flexible tools in demography that can accomplish this task and for which a large set of examples exists [28–31]. The discrete state space of stage-structured models provides computational tractability and these models can be incorporated into statistical frameworks to account for various epistemic and aleatory sources of uncertainty.

### Stage-structured matrix projection model

Using a seasonal, two-stage model for adults and first-year individuals can effectively capture the demography of most cave-hibernating bat species hibernating in winter, roosting in summer, and migrating in spring and fall [15, 18]. The model tracks females, and offspring production is assumed to conform to a 1:1 sex ratio. This arrangement means that the input starting abundance (in units of total bats of the population, males plus females) is halved before projection, and then the projected abundance results are doubled in the output. Spatial structure is not explicitly accommodated so the modeled population is assumed to be closed; however, it could represent a population at any spatial scale given certain assumptions.

Let $\textbf{x}(t) = (x_J, x_A)_t^T$ be the population abundance vector at time *t*, where $^T$ denotes the transpose of a vector, $x_J$ is the abundance of first-year individuals (juveniles) and $x_A$ is the abundance of adults. The demographic model projects the population abundance at time *t* to time $t+1$ by multiplication with the $2\times 2$ projection matrix *A* via the equation $\textbf{x}(t+1) = A \textbf{x}(t)$.

**Fig. 1:**
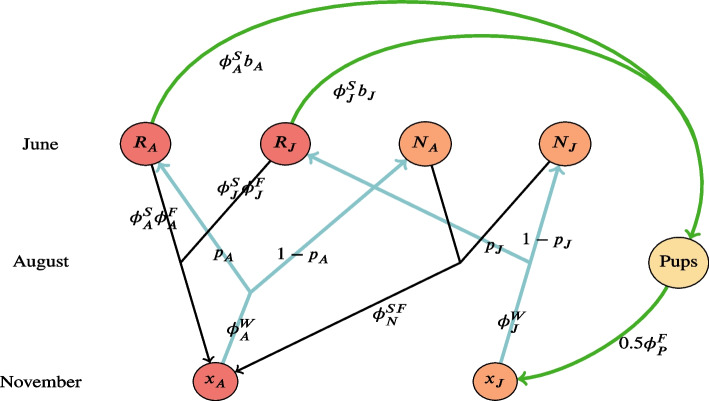
The seasonal transitions of the demographic model are represented in this hibernating bat species lifecycle diagram [32]. Bottom nodes indicate the population during winter/November, the top nodes during summer/June before pups are birthed, the middle node and vital rates indicate the transition from summer to fall to winter (black lines). The red nodes indicate the adult population, the orange nodes the first-year/juvenile population, and the yellow node the pup population. $x_A$ and $x_J$ are the total population of adults and juveniles, respectively. Survival rates are indicated by the letter $\phi$ with superscripts corresponding to seasons winter, spring, summer and fall (*W*, *G*, *S*, *F*) and superscripts corresponding to life stage (adult *A*/juvenile *J*/pup *P*). During the transition from winter to summer, the total population is split into reproducing (*R*) and nonreproducing (*N*) groups (with probabilities $p_A$ and $p_J$ for adults and juveniles), indicated by the light blue lines. Reproducing individuals producing offspring with breeding success rates ($b_A, b_J$) can then produce pups (green lines)

The projection matrix *A* is defined by the life cycle of the organism, depicted in Fig. 1. We construct a seasonal model, where superscripts W, G, S, and F on vital rates and matrices denote winter, spring, summer, and fall, respectively. Survival rates are denoted by $\phi$, reproductive propensity rates by *p*, and breeding success rates by *b*. For the fall, winter, and spring seasons, the subscripts on the vital rates denote whether they correspond to first-year/juvenile individuals (J), adults (A), or pups (P). Pups are born at the end of summer and if they survive through the fall become juveniles. During the summer the population is split into reproductive and non-reproductive groups, and in this case J represents breeding juveniles, A represents breeding adults, and N represents the combination of non-breeding adults and first-year individuals. With these conventions, the projection matrix equation can be written as follows

1 $$\begin{aligned} \left( \begin{array}{c} x_J \\ x_A \end{array}\right) _{t+1} = \left( \begin{array}{cc} 0.5\phi _J^W p_J \phi ^S_J b_J \phi _P^F &{} 0.5\phi _A^W p_A \phi _A^S b_A \phi _P^F \\ \phi _J^W p_J \phi ^S_J \phi _J^F + \phi _J^W (1-p_J) \phi _N^{SF} &{} \phi _A^W p_A \phi ^S_A \phi _A^F + \phi _A^W (1-p_A) \phi _N^{SF} \end{array}\right) \left( \begin{array}{c} x_J \\ x_A \end{array}\right) _t. \end{aligned}$$

The top left and right entries in Eq (1) represent the reproductive output from juvenile and adult bats, respectively. The bottom left entry represents the juveniles surviving into adulthood, whereas the bottom right entry represents the adult bats surviving into the next year.

All vital rates are contained in [0, 1], except fecundities ($b_J$ and $b_A$) when the annual pups per litter of a species exceed one. For example, *Myotis spp.* most often have one pup whereas *Perimyotis subflavus* more typically have two. This parameter is the only vital rate varying among different cave-hibernating bat species.

We define the following seasonal and breeding/non-breeding matrices

$$\begin{aligned} \begin{array}{l} A^W = \left( \begin{array}{cc} \phi _J^W &{} 0 \\ 0 &{} \phi _A^W \end{array}\right) \\[12pt] A^{G} = \left( \begin{array}{cc} 1 &{} 0 \\ 0 &{} 1 \end{array}\right) \end{array} \qquad \qquad \begin{array}{l} A_{N}^{S,F} = \left( \begin{array}{cc} 0 &{} 0 \\ (1-p_J) \phi _N^{S,F} &{} (1-p_A) \phi _N^{S,F} \end{array}\right) \\[12pt] A^{S}_R = \left( \begin{array}{cc} 0.5 p_J \phi ^S_J b_J &{} 0.5 p_A \phi _A^S b_A \\ p_J \phi ^S_J &{} p_A \phi ^S_A \end{array}\right) \\[12pt] A^F_R = \left( \begin{array}{cc} \phi _P^F &{} \phi _P^F \\ \phi _J^F &{} \phi _A^F \end{array}\right) . \\ \end{array} \end{aligned}$$

Then the projection matrix can be decomposed into seasonal components and direct mortality can be applied as follows

2 $$\begin{aligned} \textbf{x}(t+1) = \left[ A^{S,F}_{N} + \left( A^F_R \odot A^{S}_R\right) \right] \left[ A^{G} \left( A^{W} \textbf{x}(t) - \varvec{\tau }_{W}(t)\right) - \varvec{\tau }_{G}(t)\right] - \varvec{\tau }_{S}(t) - \varvec{\tau }_F(t), \end{aligned}$$

where $\odot$ is the element-wise product of matrices, and direct mortality is represented by $\tau _{W}(t), \tau _{G}(t), \tau _{S}(t),$ and $\tau _{F}(t)$, which are $2\times 1$ vectors to be subtracted from the population at time *t* whose two components are in units of juvenile and adults bats, respectively.

### Parameterizing the model

To project a population into the future using the demographic model, one must specify the starting abundance $(x_J, x_A)^T_{t=0}$ and the twelve vital rates making up the projection matrix *A*. Estimating stage-specific vital rates can be a difficult task, especially for elusive species such as bats. An alternative to direct estimation is to create a mapping between the growth rate of a population, $\lambda$, and the set of vital rates in the model. To create a look up table encoding this mapping, as was done in [15], one can begin with a set of vital rates, fill the projection matrix *A*, and calculate its leading eigenvalue, which corresponds to the growth rate $\lambda$. Since the mapping is not one-to-one, different combinations of vital rates can result in the same value of $\lambda$.

Environmental stressors on the population can be incorporated into the model in two ways. Direct mortality, the first method of applying a population stressor, is modeled by subtracting individuals directly from the population abundance in a given season and year; see Eq (2). This option allows for seasonal mortality events such as threats faced during spring or fall migration or habitat loss occurring in summer or winter. This approach, often relying on field records of fatalities, could quantify impacts of wind energy development, flying hazards, extreme weather, or other sources.

The second approach in our model acts by altering the vital rates in the model. This approach has been used to model the effects of WNS by decreasing adult winter survival rates according to observed patterns of disease-related decline [18]. Other rate-based estimates of stressors could be incorporated into the model in this manner as well. For instance, hypotheses pertaining to effects of diminished adult and first-year breeding success rates, reflecting observed and hypothesized effects of WNS, could also be evaluated.

The population model is deterministic with this set of input parameters, but the BatTool also includes the options of including environmental and demographic stochasticity. Environmental stochasticity is included in the model by annually perturbing the vital rates by a user-defined amount. The annual vital rates are drawn from a uniform distribution of user-input width centered at the values prescribed in the $\lambda$ look up table. There is a safeguard applied which bounds the vital rates to the range of theoretically allowed values (between 0 and either 1 for survival and birthing success rates or the maximum fecundity allowed for each species).

After these vital rates are drawn, the projection matrix *A* is defined for each year. In addition, demographic stochasticity can be optionally included in each simulation. Demographic stochasticity reflects the discrete nature of birth and death processes by using statistical distributions to model survival and reproduction instead of treating them as deterministic. In this case, we use a binomial distribution to simulate each birth and death in the model, which assumes lifetime births by the same individual are independent events.

Finally, a carrying capacity may be included in the model to prevent unrealistic exponential growth. If the total population of adult and first-year individuals exceeds the carrying capacity, the projection matrix *A* becomes the identity matrix for that time step, before the effects of stressors are added.

Thus, the model inputs include the starting abundance and population growth rate, carrying capacity, the option of demographic stochasticity and magnitude of environmental stochasticity, and annual/seasonal stressor schedules consisting of vital rate alterations and direct mortality estimates. Hereafter the set of inputs to the demographic model and its outputs will be referred to as a scenario.

For each scenario, uncertainty is captured in the model by producing an ensemble of simulations. A range of abundance values and population growth rate values can be supplied by the user, with each simulation drawing a starting abundance and growth rate from these set ranges. Vital rates are specified via the $\lambda$ look-up table, and the stochastic projection matrix is applied to the population vector in conjunction with the specified impacts of stressors. The set of simulations for a scenario take into account the variation included in the starting abundance, growth rate, mapping to vital rates, stressors, and environmental and demographic stochasticity.

Each simulation projecting abundance into the future produces an annual time series with total population size $N(t) = x_J(t) + x_A(t)$ and the derived population growth rate $\tilde{\lambda }(t) = \frac{N(t)}{N(t-1)}$. Note that the input $\lambda$ value defining the vital rates is not necessarily the derived growth rate of a given simulation. The addition of stressors and stochasticity in the model can cause the derived growth rate $\tilde{\lambda }(t)$ to deviate from the input growth rate $\lambda$. We distinguish between the input growth rate $\lambda$ and the derived growth rate $\tilde{\lambda }$; the derived population growth rate is reported in the model output.

We let the set of simulated abundance time series for simulations $k=1,\cdots ,K$ under scenario *i* be written as $\{N_k^{(i)}(t)\}_{k=1}^K$, and the set of derived growth rates $\{\tilde{\lambda }_k^{(i)}(t)\}_{k=1}^K$. Then we can define the median population abundance ${\text {med}}_k\{N_k^{(i)}(t)\}$ and median derived annual growth rate ${\text {med}}_k\{\tilde{\lambda }_k^{(i)}(t)\}$ over simulations $k=1,\cdots ,K$ at time *t* for scenario *i*, where we have suppressed the set notation over *k*. Other quantiles and summary statistics can be defined similarly.

For each scenario, the set of simulated abundance time series can be summarized using several output metrics, including the median, mean, and user-specified confidence intervals for projected abundance and derived annual growth rate, as well as the probability of survival (percent of simulations with greater than or equal to the quasi-extinction threshold specified by the user), probability of growth (percent of simulations with greater than the starting abundance at time step 0), probability of extinction (percent of simulations with ending abundance less than the quasi-extinction threshold), and the median time to extinction.

We define the *s*-year average annual growth rate between abundance estimates *N*(*t*) and $N(t+s)$ of a given population as $\gamma (t,t+s) = \left( \frac{N(t+s)}{N(t)}\right) ^{\frac{1}{s}}$. We also consider two metrics to aid in comparison between scenarios. We define the percent difference in median abundance in year *t* between scenarios *i* and *j* as

$$\begin{aligned} D_{i,j}(t)=\frac{{\text {med}}_k\{N_k^{(i)}(t)\} - {\text {med}}_k\{N_k^{(j)}(t)\}}{ \frac{1}{2}({\text {med}}_k\{N_k^{(i)}(t)\}+{\text {med}}_k\{N_k^{(j)}(t)\})}, \end{aligned}$$

and define the difference in median derived growth rate between scenarios *i* and *j* as

$$\begin{aligned} \ell _{i,j}(t) = {\text {med}}_k\{\tilde{\lambda }_k^{(i)}(t)\} - {\text {med}}_k\{\tilde{\lambda }_k^{(j)}(t)\}. \end{aligned}$$

**Fig. 2:**
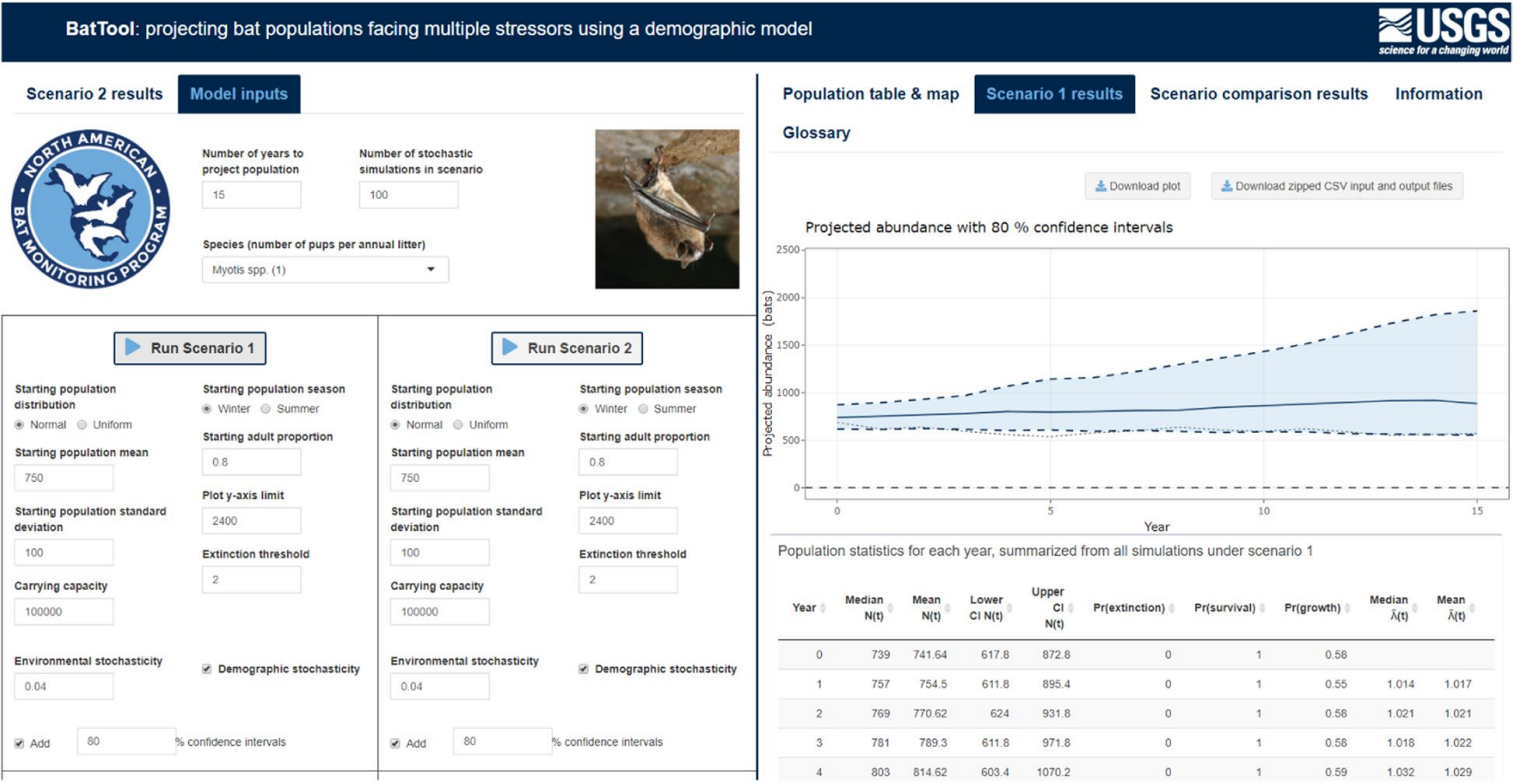
Hosted at https://rconnect.usgs.gov/battool [24, 25], the BatTool application is shown here with the model inputs tab in the left panel and the scenario 1 results tab in the right panel. The information tab on the far right provides instructions, a description of the application, and model definitions, and the population table & map tab allows the user to upload their own bat population data containing model inputs (neither shown here). Logo credit: U.S. Geological Survey; Department of the Interior

The BatTool application gives the user the ability to control all model inputs we have discussed, some of which are shown in the left panel of Fig. 2, and compare scenarios with different model inputs. Instructions for using the BatTool application can be found in the User’s guide section. The BatTool R package is available on GitLab at https://code.usgs.gov/umesc/wthogmartin/BatTool with support for installation via R and the BatTool application is hosted publicly at https://rconnect.usgs.gov/battool [24, 25].

### User’s guide

The BatTool R package [24, 25] can be installed from GitLab within R using the command remotes::install_git(“https://code.usgs.gov/umesc/wthogmartin/BatTool.git”) and friends. The package can then be loaded with library(BatTool).

The Shiny application can be launched using the function runBatTool(). Within the application, pressing either Run Scenario button will initiate the demographic model to project the starting population abundance into the future using an ensemble of simulations, with the results summarized into a plot and table, shown in the right panel of Fig. 2.

There are a handful of primary functions in the R package for command line use. The run_demographic_model() function projects an ensemble of simulations given abundance, growth rate, stressors and stochasticity model inputs, corresponding to pressing the Run Scenario buttons in the Shiny application. The project_population() function produces one simulation consisting of an abundance time series, and is used within run_demographic_model(). The summary_stats() function summarizes the output into summary statistics over time while pop_plot() creates a plot using the simulations from run_demographic_model() (the table and plot in the app are slight variations of the output from these functions).

After launching the app, the user can find a description of the model, definitions of all model inputs and outputs, instructions for saving and loading data, and more in the Information tab in the right panel.

Once the Run Scenario button has been clicked, the Scenario 1/2 results tabs will populate with a table of summary statistics and a plot of the projected median abundance with uncertainty bounds. The model inputs and outputs for one sample simulation are also summarized and plotted (dashed black line); in addition, the projection matrix and its eigendecomposition for the simulation are displayed. The Scenario comparison results tab contains a summary of the difference between scenario 1 and scenario 2 simulations.

All model inputs and outputs can be saved as CSV files contained in a ZIP file after the model has been run. The saved CSVs containing the direct mortality and white-nose syndrome vital rate reductions can then be uploaded into the app for future use. The projection plot can additionally be saved as a PNG within the app.

Finally, the Population table & map tab gives the user the option to try the BatToolPro version of the Shiny application. Clicking the checkbox loads a sample population table where each row corresponds to a population and contains model inputs, including a unique pop_name identifier, a range of abundance and growth rate values, the year of arrival of WNS, and optionally latitude and longitude values for the leaflet map displayed in the app [33].

The starting point for using BatToolPro is to click a row in the population table which selects a population to be modeled and updates the model inputs with the corresponding values in the table. The sample population table consists of fictitious populations that makes it easy for the first-time user to utilize the application. The R package includes this sample CSV (the table initialized in the app) that can be used as a template for the user to format their data for upload within the app.

The application is initialized with a set of default starting parameters arbitrarily chosen to serve as a template, so that the user can immediately press either Run Scenario button to run and produce results from the demographic model. The choice of the distribution of the starting growth rate is the most critical, determining the sets of vital rates that are possible for simulations.

This tool is flexible enough to model the population dynamics of any North American bat. The user can upload their estimates on the species of their interest. But, because the tool infers vital rates from growth rates there is inherently parametric uncertainty. It is up to the user to employ knowledge of the system to anchor growth rates and therefore vital rates to reasonable values.

Growth rates and vital rates vary among populations and can be difficult to estimate due to data limitations. Estimates are often made by aggregating populations into regional spatial scales, pooling data and thus hopefully making estimates more robust. However, even when assessing the growth rate across the entire species range there is uncertainty in estimates, and estimates change over time and as more data are collected. For these reasons, we do not include estimates of growth rates of real populations or metapopulation aggregates in the application, but inquiries about obtaining data on North American bats can be made to the North American Bat Monitoring Program.

Version >2.0.0 of the BatTool R package incorporates a number of changes to the command line tools and graphical user interface described in [18]. Model functionality is increased in two ways. First, the model now allows the input of a range of starting abundance values (akin to the $\lambda$ input), incorporating another possible source of uncertainty in the model. Second, the user now has the ability to apply female take to the first-years in the population, in addition to the adult population as was available previously. The Shiny application has several new features, providing additional user control of model settings within a more user-friendly interface. The population table is now visible with selectable rows that fill-in the model inputs. Both the population table as well as all stressor impacts can be uploaded with CSV files. Reporting of model results is improved by including tables of annual metrics directly in the application and reporting a greater range of metrics relevant to conservation decisions. In addition to tables and plots for the two scenarios individually, plots and metrics are included for the difference between the two scenarios, avoiding the need for manual post-processing. Finally, for computationally intensive scenario analyses a progress bar is displayed when running many simulations.

## Results

In this case study we demonstrate a typical workflow using the BatTool which can serve as a user template. Our goal is to investigate the potential impacts of a hypothetical wind farm in the Midwestern United States on several regional bat populations, specifically four bat species of special conservation concern whose ranges overlap with the energy generation site (*Myotis lucifugus, M. septentrionalis, M. sodalis*, and *Perimyotis subflavus*).

Taking advantage of high wind resources in the area, east-central Illinois and west-central Indiana have concentrations of existing and new wind energy development projects that may pursue incidental take coverage for listed bat species. As many cave bat species migrate between winter hibernacula situated to the south and summer breeding grounds to the north, a wind farm in this area may be a concern for these declining species.

Thus, proponents whose projects are likely to result in the take of species covered under the ESA may seek an Incidental Take Permit (ITP) under Section 10(a)(1)(B) of the ESA in consultation with USFWS [2]. The applicant submits a Habitat Conservation Plan (HCP) that includes a strategy to offset the potential negative impacts of the project, e.g. by mitigating harm done to listed species and by conserving or restoring habitat. The USFWS analyzes and describes the potential impacts of the proponent’s HCP through a National Environmental Policy Act (NEPA) document and a Biological Opinion before issuing a permit decision [34]. An important component of the Biological Opinion is a quantitative analysis evaluating the potential population-level impacts of imminent stressors, in this case wind turbines posing a flying hazard to bats.

**Fig. 3:**
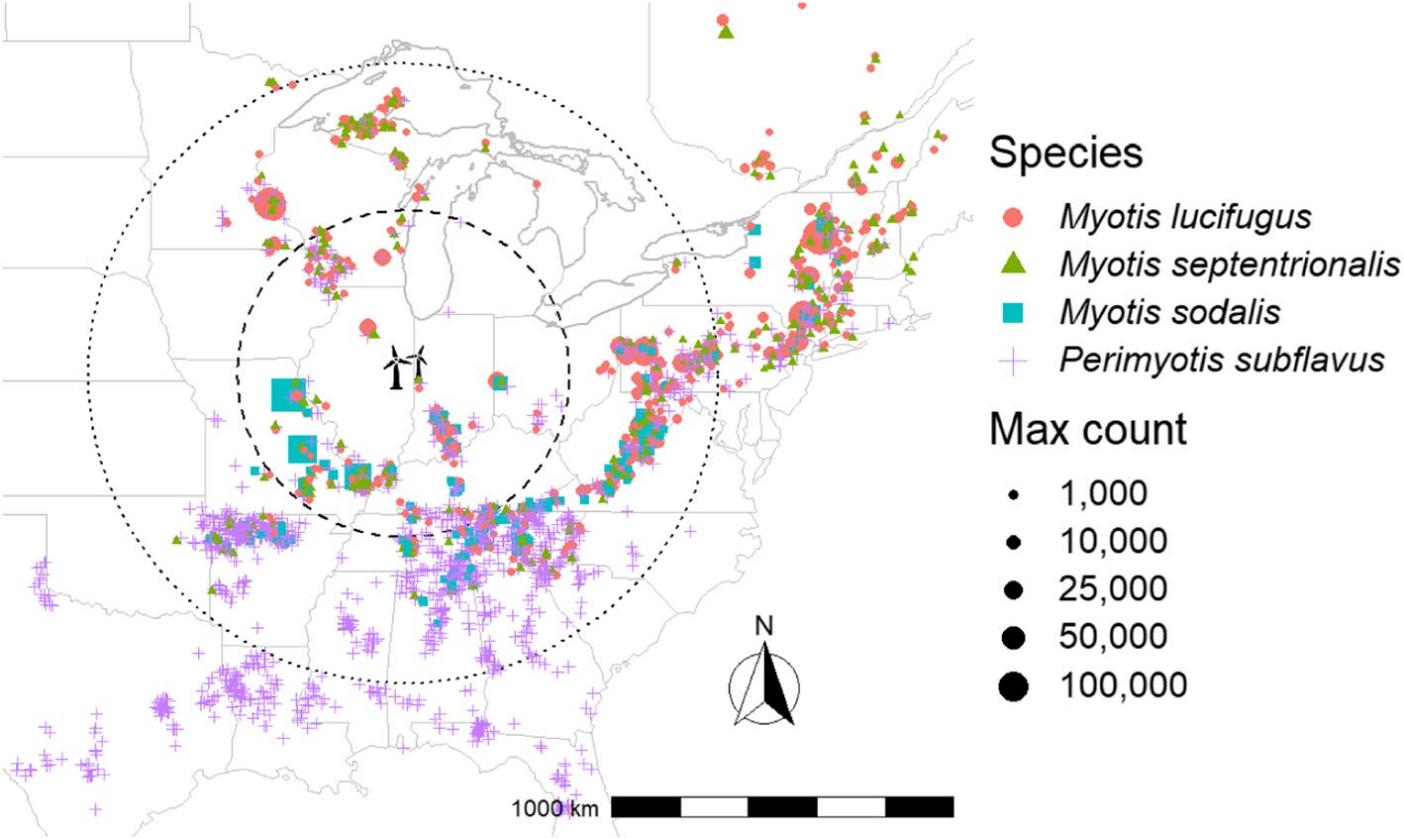
Concentric circles are the spatial catchment areas for the four regional bat populations, defined using estimated migration radii centered around a hypothetical wind farm (black wind turbine icon). The dashed inner circle defines the three *Myotis spp.* regional populations, and dotted outer circle defines the *Perimyotis subflavus* population. Approximate locations of known winter hibernacula are distinguished by color for the four bat species, and the size of the symbol indicates the maximum observed winter count at that site for data collected between 1979 and 2021 [49]. Only sites with at least one count greater than 5 bats are shown here

There are no known winter hibernacula in the immediate vicinity of the hypothetical wind farm shown in Fig. 3, so it is reasonable to assume that most impacts to these species, were they to occur, will occur during migration. Thus, we demarcated the populations of interest for each species using a spatial catchment area, defined using estimated species-specific migration radii. Estimates of the average and maximum migration distances of these species vary widely; we used four times the average migration radii of 119.69 kilometers and 227 kilometers for *Myotis spp.* and *Perimyotis subflavus*, respectively, estimated from previous studies as well as banding data [35–48].

The North American Bat Monitoring Program (NABat) has collated contributions of winter colony counts from many partners [50]. A count is comprised of a count of one or more species of bats in a winter colony (hibernacula) at a given point in time. This collection of data allows for robust estimation of species population status and assessment of the effects of stressors on species health [1]. To construct the regional populations we analyzed, we collected the set of all known winter hibernacula sites in the NABat database within 479 kilometers (*Myotis spp.*) and 908 kilometers (*Perimyotis subflavus*) of the hypothetical wind energy generation site [49]. While not strictly a closed population, modeling a large regional population more closely approximates this assumption required by the BatTool demographic model. Colony counts in the data set were divided based on whether there were three or more historical observations between 1979-2021. For each of the four species, abundance and growth rates were estimated for each site within the spatial catchment areas using Bayesian hierarchical models (if sufficiently sampled over time, i.e., >3 observations); time series were simulated from these models 600 times to characterize potential uncertainty in unobserved abundance. For less well sampled sites, we used log interpolation to predict a single abundance estimate over time. For each simulation, we then summed the estimated abundance of all sites within the spatial catchment area (for each species), yielding 600 simulations of abundance over time for each of the regional populations. The 2021 estimates of abundance were used as the starting abundance values in the demographic projection model.

WNS caused severely declining growth rates in many populations of all four of the species we considered since its presumed arrival in North America in winter 2007 [1], thus, using growth rates from the recent history of decline will project the population to extirpation. Instead, we assumed that these regional populations, which we estimated started experiencing the effects of WNS around 2010-2012, returned to growth rates experienced before the arrival of WNS. Thus, we estimated the pre-WNS population growth rates, and did not include any WNS impacts to vital rates. To estimate the regional population growth rates before the arrival of WNS, we used the set of annual growth rates calculated from the 600 simulations of abundance over time for each of the regional populations from 1979-2009. For each of the four species’ regional populations, we use the first and third quartiles of the set of annual growth rates as the (pre-WNS) starting population growth rate interval in the BatTool. The median estimates of starting abundance (with 90% CI) and growth rate (with interquartile range) for the four regional species populations are given in the third and fourth column of Table 1.

**Table 1:** Model inputs and output metrics for the four species regional populations each under three different mortality scenarios. Columns three and four show the starting abundance and growth rate distributions, distributions denoted by $[\cdot ]$. Entries in parentheses contain the 5, 50, and 95% quantiles of simulations in the given year. Columns five and six contain the ending abundance distribution and the 20-year cumulative growth rate. Columns seven and eight contain the relative abundance difference and absolute growth rate difference at the end of the simulation period between the baseline and both mortality scenarios

Species	Scenario	Starting abundance $[N_k(0)]$	Starting growth rate $[\lambda _k]$	Ending abundance $[N_k(20)]$	Avg. annual growth rate $\gamma (0,20)$	Rel. abundance difference $D_{i,0}(20)$	Abs. growth rate difference $\ell _{i,0}(20)$
*Myotis lucifugus*	Baseline	(11578, 12142, 12465)	(0.978, 0.987, 0.999)	(7611, 9476, 11763)	(-0.022, -0.012, -0.002)		
*Myotis lucifugus*	Expected	(11578, 12142, 12465)	(0.978, 0.987, 0.999)	(7445, 9122, 11319)	(-0.023, -0.014, -0.003)	(-0.316, -0.02, 0.282)	(-0.033, -0.001, 0.029)
*Myotis lucifugus*	Authorized	(11578, 12142, 12465)	(0.978, 0.987, 0.999)	(6997, 8546, 10792)	(-0.027, -0.017, -0.006)	(-0.399, -0.102, 0.24)	(-0.04, -0.006, 0.027)
*Myotis septentrionalis*	Baseline	(1701, 1779, 1864)	(0.964, 0.977, 0.994)	(810, 1121, 1586)	(-0.038, -0.023, -0.006)		
*Myotis septentrionalis*	Expected	(1701, 1779, 1864)	(0.964, 0.977, 0.994)	(746, 1095, 1494)	(-0.043, -0.023, -0.009)	(-0.543, -0.044, 0.391)	(-0.076, -0.007, 0.054)
*Myotis septentrionalis*	Authorized	(1701, 1779, 1864)	(0.964, 0.977, 0.994)	(554, 845, 1260)	(-0.057, -0.036, -0.017)	(-0.821, -0.281, 0.223)	(-0.094, -0.022, 0.052)
*Myotis sodalis*	Baseline	(192412, 192920, 193485)	(0.902, 0.934, 0.991)	(26356, 63517, 145615)	(-0.095, -0.054, -0.014)		
*Myotis sodalis*	Expected	(192412, 192920, 193485)	(0.902, 0.934, 0.991)	(26004, 60693, 139650)	(-0.095, -0.056, -0.016)	(-1.18, 0.028, 1.086)	(-0.067, -0.001, 0.062)
*Myotis sodalis*	Authorized	(192412, 192920, 193485)	(0.902, 0.934, 0.991)	(26454, 60784, 143977)	(-0.095, -0.056, -0.015)	(-1.188, -0.035, 1.114)	(-0.061, -0.003, 0.064)
*Perimyotis subflavus*	Baseline	(54228, 55026, 55781)	(0.991, 1, 1.01)	(43685, 52544, 65121)	(-0.012, -0.002, 0.008)		
*Perimyotis subflavus*	Expected	(54228, 55026, 55781)	(0.991, 1, 1.01)	(43060, 53587, 64186)	(-0.012, -0.002, 0.008)	(-0.295, 0.002, 0.309)	(-0.025, 0, 0.026)
*Perimyotis subflavus*	Authorized	(54228, 55026, 55781)	(0.991, 1, 1.01)	(42912, 52873, 64975)	(-0.012, -0.002, 0.008)	(-0.306, 0.004, 0.301)	(-0.024, 0, 0.025)

Direct mortality of bats in the form of collision with wind turbines is the primary stressor in the scenarios we investigated here. We compared three wind mortality scenarios to assess the potential impacts of wind development, a) the expected mortality from the project, b) the authorized (worst-case) mortality from the project, as well as c) a baseline scenario where no direct mortality is applied. The expected and authorized mortality data were provided by USFWS and were calculated using existing carcass recovery data from a real site. Mortality estimates are given in Table 2. See [51] for an overview of methods used to estimate bird and bat fatality rates from carcass recovery data. USFWS uses the term take, which is defined in Section 3(18) of the Federal Endangered Species Act as "to harass, harm, pursue, hunt, shoot, wound, kill, trap, capture, or collect, or to attempt to engage in any such conduct." We consider all take to result in individuals which no longer contribute to future generations through reproduction, which is effectively mortality in the model.

**Table 2:** The expected and authorized adult take (mortality) scenarios caused by the hypothetical wind farm, for each the four bat species. Estimates are in units of the number of adult individuals killed in the given season

Mortality scenario:	Expected take				Authorized take			
Species	Winter	Spring	Summer	Fall	Winter	Spring	Summer	Fall
*Myotis lucifugus*	0	0.320	1.280	3.400	0	2.540	9.940	26.520
*Myotis septentrionalis*	0	0.040	0	1.960	0	0.240	0	13.760
*Myotis sodalis*	0	0.020	0	1.480	0	0.120	0	7.380
*Perimyotis subflavus*	0	0.460	1.780	4.760	0	0.780	3.060	8.160

Previous work analyzing variation in long-term time series of *Myotis lucifugus* indicated populations often exhibit annual variation on the order of 4% of mean value in vital rates due to environmental stochasticity [4]. Since regional populations will experience less variability than one hibernacula, we fixed the model input environmental stochasticity at 0.01 for all scenarios. We also included demographic stochasticity.

Using these inputs, we utilized the BatTool to project the four regional populations 20 years into the future, equivalent to several generations of bats, from 2021 to 2041, using 600 simulations for each population and scenario. Columns five through eight of Table 1 contain several metrics summarizing the resulting simulations of the three scenarios, including the population abundance distribution at the end of simulations, the cumulative growth rate over the simulation period, and two quantities comparing the mortality scenarios to the baseline scenario.

The results for all four species are similar, showing stationary or slightly declining trajectories with average annual losses between 0–6% (Table 1 column six). All four regional populations survive the simulation period, with median and lower bounds for abundance above zero in year 2041 (Table 1 column five).

To disentangle the effects of the wind mortality from the input growth rates, we can compare the median abundance predictions among scenarios. The relative difference in median abundance $D_{i,0}(t)$ and the absolute difference in derived growth rate $\ell _{i,0}(t)$ at the end of the simulation period are shown in columns seven and eight of Table 1, where the expected and authorized mortality scenarios $i=1, 2$ are compared to the baseline scenario $j=0$. These two metrics indicate the magnitude of the impacts caused by direct mortality due to wind (quantified by the direct mortality in Table 2) because all other model inputs are equal among scenarios for each species.

For the regional *Perimyotis subflavus* and *Myotis sodalis* populations, some of the median population abundance differences are zero or positive, even with the addition of the wind take stressor. Clearly, bat mortality attributed to the wind energy facility cannot create a population increase (in the absence of some sort of density-dependent response, which we do not incorporate). That we see a positive effect on the population indicates mortality from wind energy generation is negligible in this case, and therefore the positive effect must simply be a result of stochastic variation applied in the simulations. That is, a positive difference outcome indicates the magnitude of stochasticity in the inputs obscures any effects on mortality the wind energy facility may impose. Note the *Perimyotis subflavus* population is stationary while the *Myotis sodalis* population is declining over the projection period, yet the impacts of the single wind farm appear negligible for both.

**Fig. 4:**
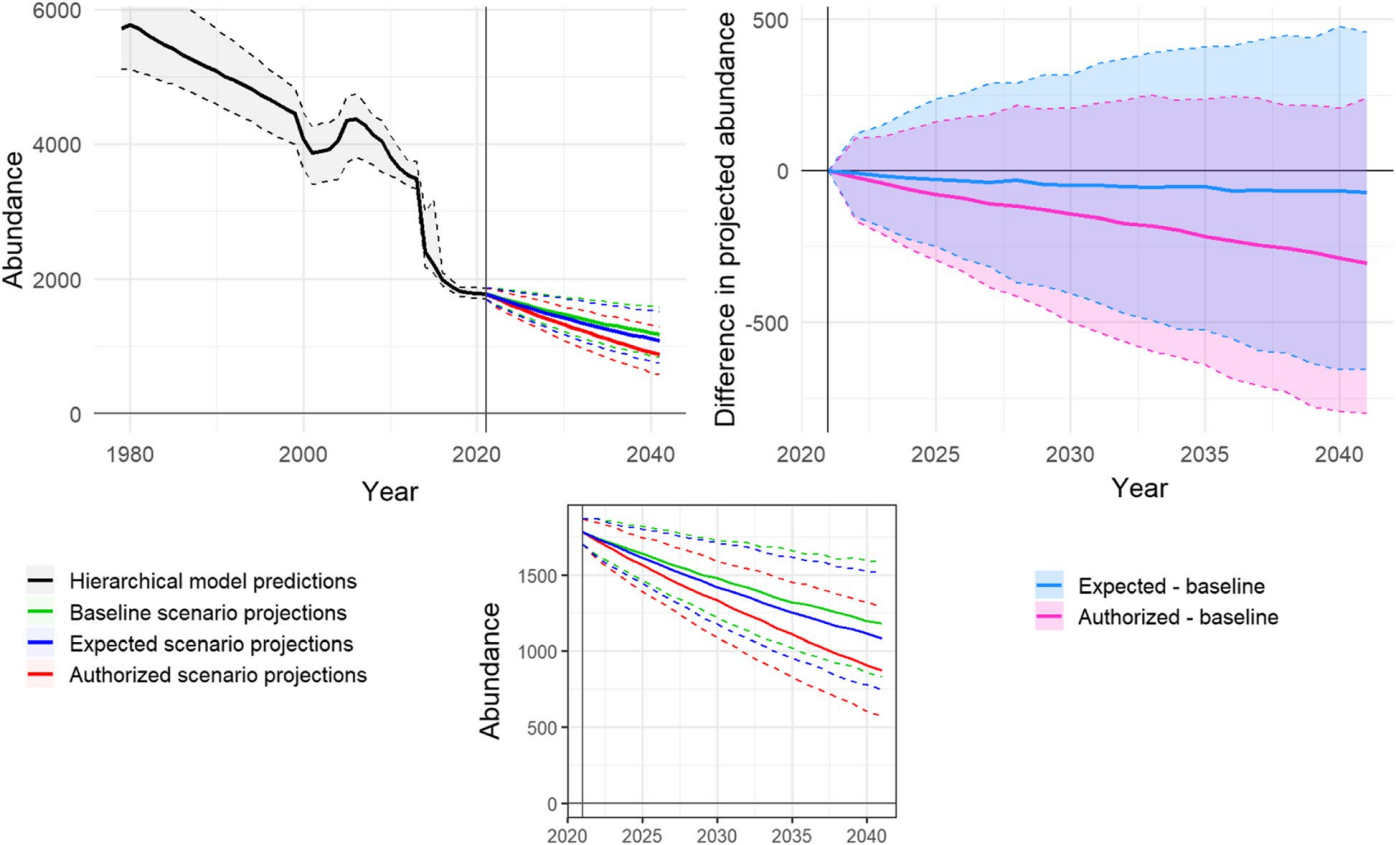
Clockwise from top left: **a** Historical modeled and future projected abundance for the *Myotis septentrionalis* regional population. The vertical line at 2021 separates abundance predictions with uncertainty from the Bayesian hierarchical model (pre-2021) and the three scenario projections from the demographic matrix model (2021-2041). The solid lines show the median and the dashed lines encompass 90% confidence intervals of simulations. **b** Simulation differences between the baseline scenario and mortality scenarios for *Myotis septentrionalis*. Solid line shows the median of simulation differences, and the dashed lines encompass 90% confidence intervals of simulation differences (y-axes in units of bats). **c** Inset enlarging the future projected abundance under three scenarios

Conversely, for *Myotis lucifugus* there is a -2– -10.2% median difference between the ending population abundance compared to the baseline scenario, and a -0.1– -0.6% median difference in ending growth rate. The impacts of wind mortality are even more severe for the *Myotis septentrionalis* population, which showed a -4.4– -28.1% median difference between the ending population abundance compared to the baseline scenario, and a -0.7– -2.2% median difference ending growth rate. An example graphical depiction of the difference in projected population trajectories between baseline and mortality scenarios for the *Myotis lucifugus* regional population from 2021 to 2041 are shown in Fig. 4.

Note that all uncertainty intervals about estimates of $D_{i,0}(t)$ and $\ell _{i,0}(t)$ overlap zero. This criterion is often used as a test of significance; in our case it is a reflection of the large amount of parametric uncertainty in the input parameters. If stochasticity or the widths of the starting abundance and growth rate distributions were reduced, the widths of the uncertainty intervals about estimates of $D_{i,0}(t)$ and $\ell _{i,0}(t)$ would be reduced similarly. It is clear by the skew in the uncertainty intervals that the Authorized *Myotis lucifugus* and Expected and Authorized *Myotis septentrionalis* scenarios intervals would not overlap zero if input uncertainty were sufficiently reduced.

On the other hand, increasing input stochasticity will often increase the widths of uncertainty intervals. To assess sensitivity of the results to stochasticity, we ran the case study with identical input parameters but heightened environmental stochasticity to 5% instead of 1%. This change caused the widths of the *Myotis lucifugus* and *Perimyotis subflavus* uncertaintry intervals to increase by about 100% for all scenarios, to increase by about 50% for all *Myotis septentrionalis* scenarios, and to cause a negligible increase for all *Myotis sodalis* scenarios (columns 5-8 of Table 3). This result corroborates our assessment of the smaller influence of stochasticity for the *Myotis sodalis* and *Perimyotis subflavus* scenarios compared to the *Myotis lucifugus* and *Myotis septentrionalis* scenarios.

**Table 3:** Case study results with identical input parameters except using 5 percent stochasticity instead of 1 percent

Species	Scenario	Starting abundance $[N_k(0)]$	Starting growth rate $[\lambda _k]$	Ending abundance $[N_k(20)]$	Avg. annual growth rate $\gamma (0,20)$	Rel. abundance difference $D_{i,0}(20)$	Abs. growth rate difference $\ell _{i,0}(20)$
*Myotis lucifugus*	Baseline	(11578, 12142, 12465)	(0.978, 0.987, 0.999)	(5397, 8034, 12051)	(-0.04, -0.02, -0.001)		
*Myotis lucifugus*	Expected	(11578, 12142, 12465)	(0.978, 0.987, 0.999)	(5161, 7867, 11781)	(-0.042, -0.021, -0.002)	(-0.601, -0.013, 0.558)	(-0.101, 0.001, 0.094)
*Myotis lucifugus*	Authorized	(11578, 12142, 12465)	(0.978, 0.987, 0.999)	(4540, 7359, 11251)	(-0.047, -0.024, -0.003)	(-0.702, -0.083, 0.505)	(-0.103, -0.009, 0.094)
*Myotis septentrionalis*	Baseline	(1701, 1779, 1864)	(0.964, 0.977, 0.994)	(598, 989, 1508)	(-0.053, -0.029, -0.008)		
*Myotis septentrionalis*	Expected	(1701, 1779, 1864)	(0.964, 0.977, 0.994)	(520, 908, 1448)	(-0.059, -0.033, -0.01)	(-0.731, -0.089, 0.525)	(-0.12, -0.01, 0.102)
*Myotis septentrionalis*	Authorized	(1701, 1779, 1864)	(0.964, 0.977, 0.994)	(376, 714, 1288)	(-0.074, -0.045, -0.017)	(-0.986, -0.319, 0.438)	(-0.148, -0.027, 0.093)
*Myotis sodalis*	Baseline	(192412, 192920, 193485)	(0.902, 0.934, 0.991)	(22143, 54814, 130304)	(-0.103, -0.061, -0.02)		
*Myotis sodalis*	Expected	(192412, 192920, 193485)	(0.902, 0.934, 0.991)	(21436, 52971, 129477)	(-0.104, -0.063, -0.02)	(-1.159, -0.009, 1.139)	(-0.104, -0.002, 0.102)
*Myotis sodalis*	Authorized	(192412, 192920, 193485)	(0.902, 0.934, 0.991)	(21508, 54776, 133985)	(-0.104, -0.061, -0.018)	(-1.191, 0.013, 1.16)	(-0.11, -0.002, 0.109)
*Perimyotis subflavus*	Baseline	(54228, 55026, 55781)	(0.991, 1, 1.01)	(23573, 38763, 66287)	(-0.042, -0.017, 0.01)		
*Perimyotis subflavus*	Expected	(54228, 55026, 55781)	(0.991, 1, 1.01)	(24625, 38224, 64399)	(-0.039, -0.018, 0.008)	(-0.699, -0.011, 0.693)	(-0.093, 0.003, 0.094)
*Perimyotis subflavus*	Authorized	(54228, 55026, 55781)	(0.991, 1, 1.01)	(23790, 39055, 64558)	(-0.041, -0.017, 0.008)	(-0.705, 0.011, 0.699)	(-0.096, 0.002, 0.086)

Finally, we assessed the sensitivity of the case study results to the size of the spatial catchment area, where we used five (instead of four) times the average migration radii to define the population of interest. Clearly, changing the population of interest is bound to change the results. Table 4 shows all populations increased in size, but growth rates remained largely the same. These changes in inputs caused little change in the results for the *Myotis sodalis* and *Perimyotis subflavus* scenarios, but they mitigated the impacts (decreases in ending population and growth rate) up to 50% in the *Myotis lucifugus* and *M. septentrionalis* scenarios, according to the $D_{i,0}(t)$ and $\ell _{i,0}(t)$ metrics.

**Table 4:** Case study results with identical input parameters except using 5 times migration radii instead of 4 times

Species	Scenario	Starting abundance $[N_k(0)]$	Starting growth rate $[\lambda _k]$	Ending abundance $[N_k(20)]$	Avg. annual growth rate $\gamma (0,20)$	Rel. abundance difference $D_{i,0}(20)$	Abs. growth rate difference $\ell _{i,0}(20)$
*Myotis lucifugus*	Baseline	(14064, 14645, 14975)	(0.978, 0.987, 0.997)	(9318, 11143, 13645)	(-0.022, -0.013, -0.003)		
*Myotis lucifugus*	Expected	(14064, 14645, 14975)	(0.978, 0.987, 0.997)	(9049, 11121, 13568)	(-0.023, -0.013, -0.004)	(-0.267, -0.008, 0.271)	(-0.03, 0, 0.03)
*Myotis lucifugus*	Authorized	(14064, 14645, 14975)	(0.978, 0.987, 0.997)	(8555, 10479, 12872)	(-0.026, -0.016, -0.006)	(-0.365, -0.054, 0.21)	(-0.035, -0.004, 0.028)
*Myotis septentrionalis*	Baseline	(1942, 2021, 2104)	(0.965, 0.977, 0.994)	(926, 1304, 1822)	(-0.039, -0.021, -0.006)		
*Myotis septentrionalis*	Expected	(1942, 2021, 2104)	(0.965, 0.977, 0.994)	(866, 1230, 1730)	(-0.041, -0.024, -0.008)	(-0.502, -0.056, 0.425)	(-0.06, -0.004, 0.062)
*Myotis septentrionalis*	Authorized	(1942, 2021, 2104)	(0.965, 0.977, 0.994)	(686, 1029, 1478)	(-0.052, -0.033, -0.016)	(-0.726, -0.233, 0.257)	(-0.089, -0.016, 0.048)
*Myotis sodalis*	Baseline	(200211, 200742, 201356)	(0.912, 0.936, 0.986)	(33683, 67695, 137337)	(-0.085, -0.053, -0.019)		
*Myotis sodalis*	Expected	(200211, 200742, 201356)	(0.912, 0.936, 0.986)	(32943, 67394, 137224)	(-0.086, -0.053, -0.019)	(-0.965, 0, 0.962)	(-0.052, 0.001, 0.055)
*Myotis sodalis*	Authorized	(200211, 200742, 201356)	(0.912, 0.936, 0.986)	(34145, 67521, 140821)	(-0.085, -0.053, -0.018)	(-1.027, 0.035, 0.976)	(-0.053, 0, 0.057)
*Perimyotis subflavus*	Baseline	(59870, 60644, 61400)	(0.991, 1, 1.009)	(48387, 59391, 71603)	(-0.011, -0.001, 0.008)		
*Perimyotis subflavus*	Expected	(59870, 60644, 61400)	(0.991, 1, 1.009)	(48525, 59253, 71671)	(-0.011, -0.001, 0.009)	(-0.296, -0.005, 0.283)	(-0.024, -0.001, 0.025)
*Perimyotis subflavus*	Authorized	(59870, 60644, 61400)	(0.991, 1, 1.009)	(48604, 59953, 71989)	(-0.011, 0, 0.009)	(-0.29, 0.006, 0.285)	(-0.024, 0.001, 0.023)

These results are based on mortality from a single wind facility and do not account for the impacts from cumulative mortality incurred from dozens of wind facilities occurring across the range of the populations. In light of this, these results give insight into whether mitigation of the additional stressor of wind energy mortality can have an effect on the future viability of these species. These outcomes may suggest that use of feathering, reduced operations during low wind speeds, and cut-in speeds could help extend the future viability for at least some of the threatened and endangered species investigated here by lessening the number of bats annually killed by wind energy generation [11, 52]. This case study illustrates how the BatTool R package can help natural resource managers quantitatively analyze scenarios relating to ESA deliberations and the issuance of Incidental Take Permits as part of regulatory decision making.

## Discussion

In this work, we introduced the updated BatTool and utilized it in a case study to quantitatively estimate potential impacts of hypothetical wind energy development. Conservation managers have been using similar analyses for over a decade, and this work is intended to provide guidance on the BatTool demographic model, graphical user interface, and an example of their application to a real scenario. Our case study can serve as a template for future analyses of stressors on bat populations. While several methods used in the case study are not specifically part of the BatTool application and demographic model (for example, mathematical models for analyzing historical data to determine starting abundance, growth rates, and WNS impacts; spatial catchment area for determining which hibernacula are included in the regional population; aggregating site growth rates to the regional spatial scale; and methods for estimating wind farm induced mortality), these methods can serve as a template for conducting similar population viability analyses. Many modifications are possible and can be tailored to the data available for the application at hand.

Given the present and expected future growth of wind energy development, it is clear mortality associated with wind energy can have measurable impacts at regional scales. More generally, this type of quantitative analysis may inform permitting and listing decisions. The summarized results from these simulations are not immutable forecasts of the population trajectory, however, but rather a glimpse into what would happen under the hypotheses and assumptions comprising each scenario. Projections incorporate a substantial amount of uncertainty, and we use the tool under the assumptions that the stage-structured demographic matrix projection model can capture the population dynamics of these species, the true abundance and growth rates of the population lie in the ranges specified, stressors are quantified and estimated reasonably, and aggregation using an abundance-weighted geometric mean is a reasonable way to assess trend of regional population. Our model also assumes a closed population, but we address this commonly violated assumption by modeling a large regional population.

Critically, the model and results are only as good as the data informing the population parameters. Most importantly, because they determine population vital rates, estimates of population-level trends must be representative and accurate, but can be sensitive to data quality. Thus, while the demographic model rests on a solid theoretical foundation, there is a danger that mis-specification of the user-supplied inputs may mis-characterize the impact of anthropogenic and environmental stressors, either by design, error, or by simply not knowing how precisely to specify the model. Safeguards against improper use of this tool arise from rigorous inspection by users and reviewers of input data and the resulting inferences.

In our case study, we modeled a regional population exhibiting heterogeneity in vital rates over space. Rather than individually projecting site-specific abundances within the region using estimated site-level growth rates (which are then translated to vital rates, akin to [15]), we use a regional population growth rate calculated as an abundance-weighted average of site-level growth rates. We believe the estimated uncertainty about the regional-level population growth rate produces appropriate uncertainty in vital rates for reflecting realistic regional population projections. Our use of an abundance-weighted geometric mean when aggregating the growth rates anticipates the possibility of a nonsensical population dynamic by taking account of both abundance (rather than distance or precision weighting) and the multiplicative nature of growth rates (using a geometric rather than an arithmetic mean). However, if a user is interested in investigating population dynamics at a finer spatial scale, this tool provides the means for understanding those dynamics.

Publicly available software such as the BatTool allows producers and users of science to work collaboratively to improve decision-making [53]. The BatTool makes sophisticated modeling of bat population dynamics readily accessible to a wider audience of biologists and conservation practitioners; we imagine there are pedagogical, especially inquiry-based, applications as well. Given the abundant threats facing bats worldwide, this tool may allow users to model the fate of species and support the design of strategies for biodiversity conservation: bats comprise roughly one-fifth of mammalian diversity.

## Data Availability

The BatTool R package is available for download at https://code.usgs.gov/umesc/wthogmartin/BatTool [24, 25]. The BatTool application is hosted at https://rconnect.usgs.gov/battool/. Data for the case study are contained in NABat database [49].
